# Development of ImmTOR Tolerogenic Nanoparticles for the Mitigation of Anti-drug Antibodies

**DOI:** 10.3389/fimmu.2020.00969

**Published:** 2020-05-20

**Authors:** Takashi Kei Kishimoto

**Affiliations:** Selecta Biosciences, Watertown, MA, United States

**Keywords:** nanoparticles, immune tolerance, rapamycin, regulatory T cells, anti-drug antibodies

## Abstract

The development of anti-drug antibodies (ADAs) is a common cause for treatment failure and hypersensitivity reactions for many biologics. The focus of this review is the development of ImmTOR, a platform technology designed to prevent the formation of ADAs that can be applied broadly across a wide variety of biologics by inducing immunological tolerance with ImmTOR nanoparticles encapsulating rapamycin. The induction of tolerance is antigen-specific and dependent on the incorporation of rapamycin in nanoparticles and the presence of the antigen at the time of administration of ImmTOR. Evidence for the induction of specific immune tolerance vs. general immune suppression is supported by the findings that: (1) ImmTOR induces regulatory T cells specific to the co-administered antigen; (2) tolerance can be transferred by adoptive transfer of splenocytes from treated animals to naïve recipients; (3) the tolerance is durable to subsequent challenge with antigen alone; and (4) animals tolerized to a specific antigen are capable of responding to an unrelated antigen. ImmTOR nanoparticles can be added to new or existing biologics without the need to modify or reformulate the biologic drug. The ability of ImmTOR to mitigate the formation of ADAs has been demonstrated for coagulation factor VIII in a mouse model of hemophilia A, an anti-TNFα monoclonal antibody in a mouse model of inflammatory arthritis, pegylated uricase in hyperuricemic mice and in non-human primates, acid alpha-glucosidase in a mouse model of Pompe disease, recombinant immunotoxin in a mouse model of mesothelioma, and adeno-associated vectors in a model of repeat dosing of gene therapy vectors in mice and in non-human primates. Human proof-of concept for the mitigation of ADAs has been demonstrated with SEL-212, a combination product consisting of ImmTOR + pegadricase, a highly immunogenic enzyme therapy for the treatment of gout. ImmTOR represents a promising approach to preventing the formation of ADAs to a broad range of biologic drugs.

## Introduction

The rise of biological therapies, first from natural sources and more recently from recombinant DNA technology, has heralded a revolution in medicine (1, 2). However, from early on, it was recognized that immune responses to biologic therapies could compromise the efficacy and safety of treatment (3–6). The formation of anti-drug antibodies (ADAs) can neutralize the activity or alter the pharmacokinetics and biodistribution of biologic drugs (5–7), cause hypersensitivity reactions, including life-threatening anaphylaxis (3, 5, 6), or cross-react with endogenous proteins (5, 8). Protein engineering has aided in reducing the risk of immunogenicity, but even biologics derived from human sequences, such as growth factors (8) and therapeutic monoclonal antibodies (9) can elicit ADAs resulting in late stage clinical trial failure (10, 11). Moreover, the current trend in protein design is to create novel structures, such as bispecific antibodies or chimeric proteins, which are foreign to the immune system (12). Prevention of ADAs in an antigen-selective manner would be desirable to reduce late stage clinical failure of promising novel biologics in development and to improve the safety and efficacy of existing products (13, 14). Here we describe the development of tolerogenic ImmTOR nanoparticles incorporating rapamycin that can be applied broadly to mitigate the immunogenicity of biologic therapies. This review describes the development of ImmTOR (section Development of Tolerogenic ImmTOR Nanoparticles), its putative mechanism of action (section Tolerogenic Properties of ImmTOR), and its application to various biologic therapies in animal models of disease (section Application of ImmTOR to Mitigating Immune Responses Against Biologic Therapies). For an overview of other immune tolerance technologies and ADA mitigation strategies, the reader is referred to other recent reviews on THE subject (15–21).

## Development of Tolerogenic Immtor Nanoparticles

**Figure 1:**
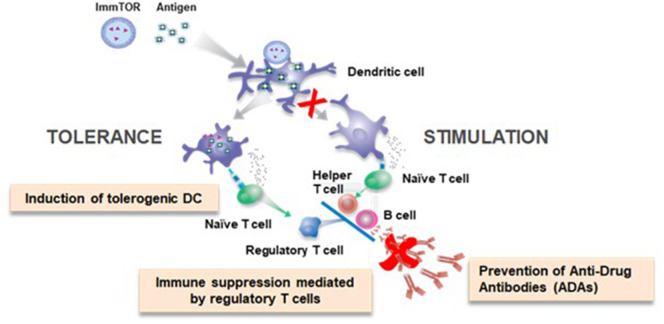
ImmTOR co-administration with antigen elicits a tolerogenic immune response.

Primary adaptive immune responses are initiated in lymphoid organs where antigen-presenting cells are poised to capture antigen and then process and present peptide fragments to T cells. Professional antigen-presenting cells, such as dendritic cells (DCs), sit at the crossroad of immune stimulation and immune tolerance (22, 23). The context in which DCs encounter antigen influences outcome of the immune response. ‘Danger signals' comprised of pathogen-associated molecular patterns (PAMPs), such as microbial toll-like receptor (TLR) agonists, or damage-associated molecular patterns (DAMPs) associated with tissue injury can activate DCs to express co-stimulatory molecules and pro-inflammatory cytokines that promote immune stimulation (24, 25). For example, traditional vaccines rely on either exogenous adjuvants or TLR agonists that are integral to microbial components of a vaccine to promote antigen-specific effector T cell responses. The purpose of an adjuvant in vaccines is to provide the pro-stimulatory context to an antigen that ensures DC activation and maturation and a robust immune response (26). Conversely immature or quiescent DCs process and present antigen that results in the formation of regulatory T cells (Tregs) (22, 23). Antigen administered in the absence of PAMPs can be tolerogenic, but there is a potential risk that the same antigen could be immunogenic if administered in a setting of inflammation. Our goal was to identify a ‘tolerogenic adjuvant' that could provide context to antigens, specifically biologic drugs, that would promote immune tolerance programming even in the face of inflammatory signals (Figure 1).

ImmTOR (previously known as SVP-rapamycin) are synthetic, biodegradable nanoparticles comprised of PLA (poly(D,L-lactide) and PLA-PEG [poly(D,L-lactide)-block-poly(ethylene-glycol)] polymers encapsulating rapamycin. We were guided by the following design criteria in developing ImmTOR: (1) use of nano-sized particles to allow for efficient targeting of DCs in lymphoid organs; (2) use of biocompatible and biodegradable polymers that have been approved for human use in multiple products, (3) use of a small-molecule immunomodulatory agent that has been validated in humans and is capable of inducing tolerogenic DCs and antigen-specific Tregs, and (4) a universal approach that could be applied to a broad range of biologic drugs in a manner that allows for immediate therapeutic benefit without the need to alter the biologic drug product. Rapamycin, alone or in combination with other immunomodulators, has been shown to have tolerogenic properties, both *in vitro* (27, 28) and *in vivo* (29–31); however, *in vivo* applications require extended daily or 3X/week administration. Our goal was to develop a technology that allows for dosing only at the time of administration of the biologic therapy.

### Why Nanoparticles?

Nanoparticles are an effective means to target DCs and other APCs in lymphoid tissues (32, 33). The immune system has evolved to filter out and interrogate nanoparticulates, which are virus size and represent a potential threat. In peripheral tissues, nanoparticulates can be endocytosed by resident DCs and myeloid cells which migrate to draining lymph nodes or can flow directly to regional lymph nodes through the draining lymphatics. Blood borne nanoparticulates are filtered out in the spleen and liver. Indeed, whole animal imaging of mice injected with fluorescent labeled ImmTOR showed accumulation of ImmTOR in the draining popliteal, iliac, and renal lymph nodes within 1 h after subcutaneous (s.c.) injection in the hind limb and similarly rapid accumulation in the spleen and liver following intravenous (i.v.) administration (34). Within the spleen, immunohistochemistry showed co-localization of ImmTOR particles with dendritic cells in the marginal zone as well as within macrophages (34). These findings were confirmed by flow cytometric analysis of splenocytes, showing a significant fraction of conventional DCs, plasmacytoid DCs, monocytes and macrophages had endocytosed fluorescent-labeled ImmTOR (34, 35). In contrast, 1% or less of CD4 T cells, CD8 T cells, B cells, and neutrophils were positive for fluorescent ImmTOR (35). These results indicate that ImmTOR leverages the natural disposition of nanoparticulates to target APCs in lymphoid organs.

### Use of PLA Polymers

ImmTOR is primarily composed of the biodegradable polymers PLA and PLA-PEG. PLA is part of the broader PLGA [poly(lactide-co-glycolide)] family of biodegradable polymers that have more than 30 years of clinical use and are formulation components in a number of approved products, including Zoladex®, Risperdal® Consta®, Vivitrol® and Lupron Depot® (36). PLA- and PLGA-based nanoparticles are hydrolyzed in an acidic environment, such as that of the endosome, and the release of the payload can be tuned for optimal activity *in vivo* (37). PLA is hydrolyzed to lactic acid, a natural metabolite that is rapidly cleared. PEG has also been widely studied in clinical trials and is also a formulation component in many approved biological products (38).

### Selection of Rapamycin

Rapamycin, a natural macrolide compound that inhibits the mammalian target of rapamycin (mTOR) pathway, has been shown to have tolerogenic properties *in vitro* (27, 28) and *in vivo* (29–31).

Thomson and colleagues demonstrated that treatment of DCs *in vitro* with rapamycin induced a tolerogenic phenotype that promoted the induction of Tregs (27). Murine bone-marrow-derived DCs propagated *ex vivo* in the presence of rapamycin express low levels of MHC class II and significantly reduced levels of co-stimulatory molecules CD40, CD80, and CD86 (27).

The mTOR pathway also differentially regulates effector T cell vs. Treg activation and differentiation (28, 39, 40). IL-2 promotes proliferation of effector T cells through activation of the JAK/STAT5 pathway and the phosphatidylinositol 3-kinase (PI3K)/Akt/mTOR pathway downstream of the IL-2 receptor. While IL-2 is a critical survival factor for Treg, it does not promote robust proliferation due to expression of PTEN, a negative regulator of the PI3K/Akt/mTOR pathway (40). The mTOR pathway promotes effector T cell expansion by regulating the metabolic switch to glycolysis, which meets the energetic requirements of rapidly proliferating cells (39). In contrast, Tregs rely on mitochondrial oxidative metabolism rather than glycolysis. Rapamycin has been shown to selectively suppress the activation of effector T cells by inhibiting the PI3K/Atk/mTOR pathway, while permitting the differentiation and expansion of Tregs (41, 42). Rapamycin is approved for the prevention of renal transplant rejection (43), but does not induce tolerance in transplantation, perhaps in part due to its use in combination with calcineurin inhibitors that inhibit both effector T cells and Tregs (44).

### Universal Approach to ADA Mitigation

**Figure 2:**
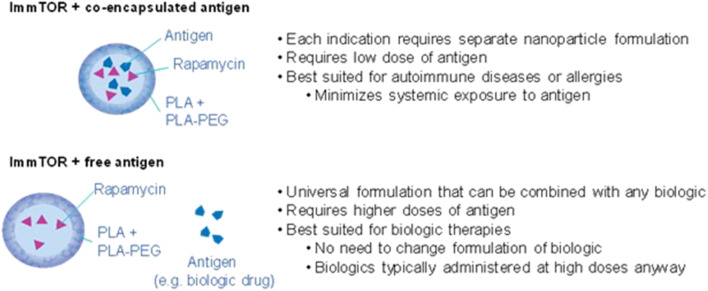
ImmTOR with co-encapsulated antigen vs. ImmTOR + free antigen.

We initially demonstrated that nanoparticles that co-encapsulated both rapamycin and antigen were effective at inducing durable antigen-specific immunological tolerance *in vivo*, including against coagulation factor VIII in a mouse model of hemophilia A (Figure 2) (34). This approach utilizes encapsulation of the biologic in the nanoparticle to induce immune tolerance with either concomitant or subsequent treatment with the free biologic to provide therapeutic activity. The advantage of this approach is that it ensures efficient co-delivery of both the rapamycin and antigen to the same antigen presenting cells. However, the disadvantage for applications involving ADA mitigation is that this approach requires encapsulation of the biologic drug, which would alter its biodistribution and activity. It is possible that ImmTOR + co-encapsulated antigen particle could be used as an initial tolerizing therapy prior to or concurrent with administration of the free biologic drug (45). However, this would still require new formulation development for each biologic drug and GMP manufacturing of both free and nanoparticle-encapsulated drug. For these reasons, the ImmTOR + co-encapsulated antigen approach may be best suited for use as tolerogenic therapies for autoimmune disease or allergies, in which minimizing systemic exposure of autoantigens or allergens, respectively, would be desirable (34, 46).

For the purpose of inhibiting ADAs, it was desirable to have a universal approach that can be applied to any biologic drug therapy without the need to optimize the nanoparticle for each specific biologic and without having to alter the biologic itself or its intended dose route or regimen. We discovered that ImmTOR particles containing only rapamycin could simply be co-administered with a free antigen to induce immunological tolerance (Figure 2) (47). As with ImmTOR particles encapsulating both rapamycin and antigen, ImmTOR particles containing only rapamycin and co-administered with free antigen were capable of inducing tolerogenic dendritic cells and Tregs that were specific to the co-administered antigen (35, 47, 48).

In the case of ImmTOR-rapamycin particles co-administered with free antigen, the ImmTOR nanoparticles show limited biodistribution to APCs in the liver and spleen, following i.v. administration (34), while the free antigen typically biodistributes broadly. However, as long as some portion of the antigen co-localizes with the APCs that take up ImmTOR particles (35), the resultant tolerogenic DCs can induce antigen-specific Tregs that, in turn, can circulate to other tissues to suppress the immune response against the target antigen. A key advantage of this approach is that the formulation of the biologic drug does not have to be altered—the biologic is simply administered as intended together with the ImmTOR-rapamycin particle. It is not necessary to physically admix the ImmTOR with the biologic prior to injection; the two components can be injected sequentially (35).

## Tolerogenic Properties of Immtor

### ImmTOR vs. Free Rapamycin

Moghimi et al. (31) reported that free rapamycin administered daily mitigated the formation of antibodies to a sub-therapeutic dose of coagulation factor VIII but not to a therapeutic dose (see section Coagulation Factor VIII for additional detail). In our hands, free rapamycin, even administered daily, did not have the same tolerizing effect as ImmTOR against a highly immunogenic antigen, keyhole limpet hemocyanin (KLH) (47). Mice immunized with 3 weekly doses of KLH and treated concurrently with either daily doses of 50 μg free rapamycin for 3 weeks (5 days per week) or with weekly doses of ImmTOR particles containing 50 μg rapamycin were tested for the generation of immune tolerance to KLH. During the treatment period, both free rapamycin and ImmTOR were similarly effective in suppressing the anti-KLH antibody response. The treated mice were then challenged with 3 weekly injections of KLH alone. The mice treated with free rapamycin + KLH developed a robust antibody response to KLH that was indistinguishable from naïve mice that had received only the three injections of KLH alone. In contrast, the animals that had been treated with ImmTOR + KLH were still seronegative after the three KLH challenge injections, even though the total rapamycin exposure was five times lower in the ImmTOR group than that of the mice treated with free rapamycin (47). These results highlight the difference between immune suppression vs. immune tolerance, where immune suppression is mediated by a drug with no lasting immunosuppressive effect after the drug is cleared. Whereas, immune tolerance is mediated by immune cells which maintain tolerance even after the drug is cleared. The basis for this difference between free rapamycin and ImmTOR is not entirely clear. *In vitro*, in a static tissue culture well, ImmTOR is not more effective than free rapamycin in inducing Tregs (unpublished observation); therefore, we hypothesize that the difference *in vivo* is related to the selective biodistribution of ImmTOR to lymphoid organs and its preferential uptake by antigen-presenting cells (34, 35). In contrast, rapamycin distributes broadly and extensively into organs and tissues (49). It is known that different doses of rapamycin are needed to inhibit phosphorylation of different mTOR substrates and in different cell types (50). It is possible that conventional dosing of free rapamycin cannot achieve the local concentration necessary to induce a robust tolerogenic phenotype in dendritic cells.

### Tolerogenic Window

The ImmTOR particles opens a tolerogenic window that is defined both temporally and spatially. Temporally the free antigen must be concomitantly administered with ImmTOR (35, 47), indicating that ImmTOR is not simply acting as a slow release formulation of rapamycin that mediates chronic immune suppression. This is consistent with findings that adoptive transfer of tolerance requires treatment of donor animals with both ImmTOR and antigen, as either alone were incapable of inducing regulatory cells capable of transferring tolerance to naïve recipients (35, 46, 48). Spatially, as noted above, fluorescent-labeled nanoparticles show restricted biodistribution to APCs in the spleen and liver following i.v. administration (34). While free antigen is expected to biodistribute broadly, the APCs that take up ImmTOR are also flooded with free antigen during the temporal window which enables peptide epitopes from the free antigen to effectively compete for presentation on MHC molecules expressed by the ImmTOR modified APCs (35). In contrast pathogen-derived antigens, which are likely to enter the body through the lung, gut or skin, will be concentrated in regional lymph nodes draining these tissues.

### *In vivo* Induction of Tolerogenic Dendritic Cells

We evaluated the ability of ImmTOR to induce tolerogenic DCs *in vivo* by treating mice with ovalbumin 323–339 peptide (OVA peptide) alone or in combination with ImmTOR (47). The next day, splenic DCs were isolated and co-cultured with OVA peptide-specific OTII T cells. The DCs isolated from animals treated with OVA peptide + ImmTOR increased the percentage of Foxp3^+^, CD25^hi^ OTII T cells, while DCs isolated from mice treated with OVA peptide alone increased the percentage of interferon-γ producing effector OTII T cells (47). These results demonstrate the ability of ImmTOR co-administered with antigen to induce tolerogenic DCs *in vivo* that are capable of promoting antigen-specific Tregs.

### *In vivo* Induction of Antigen-Specific Treg

The ability of ImmTOR to induce antigen-specific T cells *in vivo* was first demonstrated using nanoparticles encapsulating both rapamycin and OVA peptide (34). OVA peptide-specific OTII transgenic T cells were adoptively transferred into naïve mice and then treated the next day. Mice treated with ImmTOR particles containing OVA peptide and rapamycin show reduced numbers of total OTII T cells and an increased percentage of Foxp3^+^, CD25^hi^ OTII T cells compared to mice treated with nanoparticles containing OVA peptide alone. OVA peptide particles co-administered with free rapamycin actually showed the reverse trend, with a lower percentage of Foxp3^+^, CD25^hi^ OTII T cells compared to control animals treated with OVA peptide particles alone, suggesting that ImmTOR mediates fundamentally different biological outcomes than free rapamycin (34). Similar induction of OVA-specific Tregs was shown for ImmTOR particles containing rapamycin alone co-administered with free OVA peptide (47).

The use of adoptively transferred transgenic OTII cells specific for OVA may not reflect an endogenous T cell response. We assessed the ability of ImmTOR to induce endogenous antigen-specific Treg using the 2W1S peptide described by Nelson et al. (51). Mice treated with ImmTOR particles containing both 2W1S peptide and rapamycin substantially increased the number and percentage of endogenous 2W1S-specific, Foxp3^+^, CD25^hi^ Treg as detected using a 2W1S-MHC class II tetramer (46). The increased number and percentage of 2W1S-specific, Foxp3^+^, CD25^hi^ Treg were maintained even following challenge with 2W1S peptide co-administered with a potent TLR7/8 agonist or emulsified in complete Freund's adjuvant. Meliani et al. (48) also showed that ImmTOR increased the percentage of lymph node T cells with a follicular regulatory (CXCR5^+^, PD1^+^, Foxp3^+^) phenotype, which may play a key role in inhibiting germinal center B cell responses.

Another hallmark of immune tolerance is the ability to transfer tolerance from treated animals to naïve animals by adoptive transfer of immune cells. Adoptive transfer of tolerance induced by ImmTOR was demonstrated by three separate laboratories (35, 46, 48). The transfer of tolerance required treatment of donor mice with both ImmTOR and antigen; donor mice treated with ImmTOR particles containing rapamycin without co-encapsulated or co-administered antigen were unable to confer tolerance to recipient mice (35, 46). Moreover, the tolerogenic activity of ImmTOR was partially negated by depletion of CD25^+^ T cells, which are enriched for Tregs (35, 48). The inability of anti-CD25 depleting antibodies to completely restore the immune response in ImmTOR-treated animals may reflect additional mechanisms of tolerance mediated by other (non-CD25^+^) regulatory cells or simply incomplete depletion of CD25^+^ Tregs.

### Effect of ImmTOR on Effector T and B Cell Responses

#### Effector and Memory T Cell Responses

Animals treated with ImmTOR + antigen showed reduced antigen-specific T cell activation, proliferation, interferon-γ production, and *ex vivo* antigen-recall responses (47, 48). Adoptive transfer of antigen-experienced immune cells into tolerized donor mice that had previously been treated with ImmTOR + antigen were inhibited in responding to *in vivo* antigen challenge (34, 46). Similarly, recipient SJL mice treated with ImmTOR nanoparticles containing rapamycin and PLP peptide, but not with ImmTOR particles containing rapamycin without antigen, were protected from the development of experimental autoimmune encephalomyelitis following subsequent transfer of activated PLP-specific encephalitogenic T cells (46). These results provide further evidence for an induction of an antigen-specific regulatory cell population capable of inhibiting activated effector T cells.

The ability of ImmTOR to inhibit memory T cells was evaluated by immunizing donor mice with adeno-associated viral (AAV) vector and allowing memory T cells to form (48). Sixty-two days after immunization, antigen-experienced CD4 T cells were transferred into naïve recipient mice which were subsequently challenged with AAV alone or AAV + ImmTOR. The addition of ImmTOR enabled inhibition of the antibody response to AAV even in the presence of antigen-experienced memory T cells (48).

#### Effector B Cell Activation and Antibody Production

Inhibition of antigen-specific B cells was demonstrated with adoptively transferred hen egg lysozyme (HEL)-specific transgenic MD4 B cells. Treatment of mice with ImmTOR containing rapamycin and either co-encapsulated HEL or co-administered free HEL inhibited MD4 B cell activation and proliferation compared to mice treated with HEL alone (34, 47). Treatment of mice with ImmTOR + AAV inhibited the expansion of endogenous antigen-specific B cells, as determined by ELISpot analysis for both IgG- and IgM-secreting splenic B cells, without affecting the total number of B cells (48). ImmTOR treatment also strongly reduced the presence of activated germinal center B cells (34, 47, 48). Moreover, the percentage of B cells expressing an anergic or regulatory phenotype was significantly higher in animals treated with ImmTOR + antigen vs. antigen alone (47, 48).

These results are consistent with the ability of ImmTOR to inhibit antibody responses to a variety of antigens. ImmTOR was capable of completely inhibiting the formation of antigen-specific IgG1, IgG2a, IgG2b, and IgG3 antibodies in mice (35, 48). Importantly, ImmTOR also inhibited the formation of antigen-specific IgE antibodies that could potentially cause hypersensitivity responses (34). ImmTOR has shown varying activity in inhibiting an IgM response (35, 47, 48).

ImmTOR treatment had no apparent effect on pre-existing bone marrow plasma cells (35), as expected, as long-lived plasma cells do not require T cell help (52). From a safety perspective, it would be undesirable to deplete long-lived plasma cells as these cells produce protective immunity against previously encountered pathogens and vaccines.

## Application of ImmTOR to Mitigating Immune Responses Against Biologic Therapies

We have tested the ability of ImmTOR to mitigate the formation of ADAs against a variety of highly immunogenic biologic therapies with different physicochemical properties, dose routes and dose regimens (Table 1).

**Table 1:** Mitigation of antibodies against biologic therapies by ImmTOR in preclinical animal models.

**Biologic (disease)**	**Preclincal model**	**Key results**	**Reference**
Pegadricase (chronic refractory gout)	Hyperuricemia in uricase-deficient mice	• Mitigated ADA formation and enabled sustained reduction of serum uric acid in uricase deficient mice • Mitigated ADA formation and prolonged serum uricase activity in non-human primates	(47)
Adalimumab (autoimmune diseases)	Inflammatory arthritis in transgenic mice expressing human TNFα	• Sustained mitigation of ADAs even after 9 challenge injections of adalimumab alone • Improved clinical outcome as measured by arthritis score, histopathology of joints, and radiographic imaging	(47)
Coagulation factor VIII (hemophilia A)	Factor VII-deficient mice	• Sustained mitigation of ADAs even after multiple challenge injections of factor VIII alone administered over 5.5 months • Sustained blood coagulation with repeated dosing • Did not affect immune responses to other unrelated antigens administered during challenge period • Mitigated ADA response in animals previously exposed to Factor VIII	(45)
LMB-100 immunotoxin (mesothelioma)	Mesothelioma tumor in mice	• Sustained mitigation of ADAs even after 11 challenge injections of LMB-100 alone • Did not affect immune responses to other unrelated antigens administered during challenge period • Mitigated ADA response in animals previously exposed to LMB-100 • Improved survival in tumor bearing animals • Adoptive transfer of splenocytes from treated animals to naïve animals mitigates subsequent ADA formation	(35)
Acid alpha-glucosidase (Pompe disease)	Acid alpha-glucosidase-deficient mice	• More durable inhibition of ADA responses compared to animals treated with methotrexate • Higher glycogen clearance in skeletal muscles and improved motor function • No decrease in body weight compared to animals treated with methotrexate	(53)
Adeno-associated vectors (gene therapy of inherited diseases)	Human factor IX in mice, mouse models of methylmalonic acidemia and ornithine transcarbamylase deficiency	• Mitigation of anti-AAV antibodies, enabling redosing of AAV vector in mice and non-human primates • Antigen-selective to specific serotype of AAV • Inhibition of antigen-specific effector T and B cell responses • Adoptive transfer of splenocytes from treated animals to naïve animals mitigates subsequent ADA formation • Depletion of CD25+ cells partially restores immune response in ImmTOR-treated animals	(48)

### Coagulation Factor VIII

Replacement coagulation factors has been a mainstay therapy for the treatment of hemophilia patients, such as Factor VIII (FVIII) therapy for the treatment of hemophilia A and Factor IX therapy for the treatment of hemophilia B (54). The formation of neutralizing antibodies (inhibitors) against FVIII occurs in 20–30% of patients with severe hemophilia A treated with replacement factor, exposing these patients to increased risk of bleeding episodes. While bypass therapies exist, such as Factor VIIa (55) and the bi-specific emicizumab antibody (56), the development of ADAs is still a major complication for patients with hemophilia A (57).

Initially nanoparticles containing both rapamycin and FVIII were used to demonstrate the induction of durable antigen-specific immunological tolerance in a mouse model of hemophilia A (34). Zhang et al. (45) studied ImmTOR particles containing co-encapsulated FVIII and ImmTOR co-administered with free FVIII. Both strategies were effective in mitigating ADAs against FVIII, even after multiple challenge injections of FVIII alone. However, the two types of ImmTOR particles (Figure 2) were studied using different treatment regimens and different challenge injections, so the results cannot be directly compared. In the former case, hemophilia A mice received two tolerizing doses of ImmTOR + co-encapsulated FVIII followed by three additional weekly injections of the nanoparticle concurrently with 3 weekly injections of a therapeutic dose of free FVIII. The mice were then challenged with 4 injections of FVIII alone. The anti-FVIII response was effectively inhibited, with the exception of one mouse that showed high titers. Mice treated with ImmTOR + co-encapsulated FVIII showed normalized bleeding responses to repetitive treatment with FVIII (45). While effective in inhibiting the formation of total anti-FVIII IgG and anti-FVIII neutralizing antibodies, this approach utilizes encapsulation of the FVIII in the nanoparticle with either concomitant or subsequent treatment with the free FVIII (34, 45), which is not ideal for drug development (see section Universal Approach to ADA Mitigation). Thus, subsequent studies investigated ImmTOR co-administered with free factor VIII.

ImmTOR particles containing rapamycin alone co-administered with free FVIII was similarly effective and specific in mitigating the formation of ADAs (45). The advantage of this approach from a drug-development perspective is that the biologic drug is not physically altered. Five weekly co-administrations of ImmTOR particles containing rapamycin with therapeutic doses of free FVIII induced durable mitigation of ADAs that was maintained for at least 5 months despite repeated challenges of FVIII alone but did comprise the immune response to other antigens. Moghimi et al. (31) previously reported that free rapamycin administered daily (6x/week) for 1 month with factor VIII mitigated the ADA response to subsequent dosing of factor VIII alone (31). Both Zhang et al. (45) and Moghimi et al. (31) administered the same amount of rapamycin (in ImmTOR or as free drug, respectively) per dose, but due to the daily doing of free rapamycin vs. weekly dosing of ImmTOR, the cumulative rapamycin dose was 6X higher for free rapamycin than that required with ImmTOR over the same 1 month tolerizing period (31, 45). In addition, the free rapamycin had to be administered with a sub-therapeutic dose of factor VIII during the tolerizing period. A key advantage of the ImmTOR approach is being able to administer the tolerizing therapy with therapeutic doses of the biologic such that the patient can receive therapeutic benefit immediately from the beginning of therapy, without the need for a lead-in tolerization period.

Zhang et al. (45) also showed that ImmTOR + free FVIII was therapeutically efficacious in controlling the ADA response in hemophilia A mice that were pre-sensitized to factor VIII. Initially, mice that had low levels of anti-FVIII antibodies prior to the start of treatment showed an initial increase in anti-FVIII antibody levels, but the levels steadily decreased after a second course of therapy; whereas, control mice treated with empty nanoparticles + FVIII showed increasing titers over time.

### Pegylated Uricase

Humans lack endogenous uricase, an enzyme that metabolizes uric acid, and consequently can develop gout, a disease caused by deposition of urate crystals in joints and soft tissues leading to leading to painful gout flares, bone remodeling, and disability (58). Recombinant pegylated uricase has been developed as a promising therapy for the treatment of chronic gout refractory to oral therapies and has been shown to rapidly and efficiently reduce tissue urate crystal deposits (59). However, pegylated uricases are highly immunogenic tetrameric enzymes that are foreign to the human immune system. The marketed product, pegloticase, induces anti-drug antibodies (ADAs) in ~90% of patients (59, 60). The formation of high titer ADAs correlates with the loss of efficacy and increased risk of infusion reactions (59).

The addition of ImmTOR to pegadricase (formerly known as pegsiticase), a pegylated recombinant uricase derived from Candida utilis, prevented the formation of ADAs in uricase-deficient mice and enabled sustained control of serum uric acid in these hyperuricemic mice (47). Similarly, ImmTOR mitigated the immunogenicity of pegadricase and prolonged the pharmacodynamic activity of the enzyme in non-human primates. In addition to inhibiting the anti-uricase IgG response, ImmTOR also inhibited the IgM response to the pegylated enzyme (47). SEL-212, a combination product consisting of ImmTOR + pegadricase, is currently being evaluated in a Phase 2 study in patients with chronic gout refractory to oral therapies (61) (see section Clinical Translation).

### Adalimumab

Adalimumab is a monoclonal antibody directed against tumor necrosis factor-α (TNF-α) and approved for the treatment of rheumatoid arthritis, ankylosing spondylitis, Crohn's disease, ulcerative colitis, plaque psoriasis and other autoimmune diseases (62). It has been the best-selling drug for many years with annual sales approaching $20 billion. Despite being the first fully human monoclonal antibody approved by the FDA, adalimumab is highly immunogenic (9). Greater than 70% of healthy volunteers develop ADAs after a single injection (63–65). The formation of ADAs in rheumatoid arthritis patients was associated with accelerated drug clearance and poor outcomes. Only 3.9% of patients that developed ADAs experienced sustained remission, compared to 34% for patients that that did not develop ADAs (9).

Adalimumab, unlike FVIII or pegylated uricase, is administered by subcutaneous (s.c.) administration. ImmTOR administered s.c. localizes to the draining regional lymph nodes (34). We evaluated the ability of ImmTOR to mitigate the immunogenicity of adalimumab administered s.c. in a transgenic mice expressing human TNFα which spontaneously develop inflammatory arthritis. Co-administration of ImmTOR with adalimumab for 7 weekly injections mitigated the formation of ADAs that was sustained even after 9 additional weekly injections of adalimumab alone (47). Although low titers of antibodies (<1:100) developed by the end of the study, these did not appear to affect clinical outcome. The combination treatment normalized adalimumab blood levels throughout the 16 week treatment period and prevented the development of arthritis as assessed by clinical scores, histopathology, and microCT imaging.

### Immunotoxin

Recombinant immunotoxins are chimeric proteins containing a tumor-targeting antibody fragment linked to a protein toxin, such as pseudomonas exoprotein A (66). Recombinant immunotoxins have shown promising clinical activity, highlighted by the recent approval of moxetumomab dasudotox for the treatment of relapsed or refractory hairy cell leukemia (67); however, the bacterial toxin moiety is highly immunogenic, which limits the efficacy of immunotoxins in patients that do not have comprised immune systems. Pastan et al. (68) undertook the herculean task to deimmunize the pseudomonas exoprotein A toxin by painstakingly mapping antibody and T-cell epitopes through mutagenesis and functional analysis, rather than by *in silico* prediction which is prone to artifacts. While immunogenicity could be substantially reduced, it could not be fully eliminated without compromising the activity of the immunotoxin.

The Pastan group showed in preclinical studies that ImmTOR was capable of inducing immune tolerance to LMB-100, a partially de-immunized mesothelin-targeted immunotoxin being developed for the treatment of mesothelioma and other solid tumors (35). LMB-100 is administered in cycles, in which a cycle consists of three infusions of LMB-100 administered every other day at the beginning of each cycle. ImmTOR administered at the first dose of each cycle was sufficient to mitigate the ADA response, and two such cycles of treatment was sufficient to enable immune tolerance that allowed for at least three additional cycles (nine injections) of LMB-100 alone without compromising the immune response to other antigens (35). Interestingly, administering ImmTOR on the second dose of LMB-100 in each cycle (2 days after the first dose of LMB-100) was ineffective, underscoring the need to administer ImmTOR within a narrow time window of the first dose of antigen (see also section Tolerogenic Window). ImmTOR mitigated the formation of all IgG subtypes specific for LMB-100 but had no apparent effect on the IgM response. The mitigation of immunogenicity enabled repeated administration and allowed for control of tumor growth and improved survival in a mouse model of mesothelioma, even in mice that were pre-sensitized to LMB-100 prior to treatment (35). This is significant, because some patients have pre-existing antibodies that cross-react with LMB-100, presumably from prior exposure to pseudomonas bacteria (69).

The activity of ImmTOR in pre-sensitized mice was further investigated in two studies (35). In the first study, mice were sensitized with 6 doses of LMB-100 and then rested for 6 weeks prior to treatment. The sensitized animals showed low ADA titers at the time of treatment. Titers remained low following treatment with LMB-100 + ImmTOR and subsequent re-challenge with LMB-100 alone. In contrast, sensitized mice re-challenged with LMB-100 alone showed a large increase in ADA titer, characteristic of an anamnestic response. In the second study, mice were pre-sensitized with 12 injections of LMB-100 to induce high ADA titers (~10,000–30,000). Subsequent treatment with LMB-100 + ImmTOR was able to reduce titers about 5–10-fold. However, this level of reduction may not be sufficient to allow for therapeutic activity of the immunotoxin. These results suggest that ImmTOR, which targets the dendritic cell-T cell axis, may not be sufficient to mitigate high levels of pre-existing antibodies.

Mazor et al. (35) also studied the combination of LMB-100 with anti-CTLA4 or anti-OX40 checkpoint inhibitors. The checkpoint inhibition enhanced the ADA response to LMB-100, particularly in the case of CTLA4 blockade which increased anti-LMB-100 titers ~8-fold. Interestingly ImmTOR was able to inhibit the formation of ADAs in in the presence of checkpoint blockade. However, the effect of ImmTOR on the anti-tumor activity of the checkpoint inhibitors was not investigated. It is possible that different regimens would have to be explored in order to successfully combine ImmTOR with LMB-100 and checkpoint inhibitors (e.g., dosing checkpoint inhibitors after LMB-100 + ImmTOR therapy).

### Alglucosidase Alfa

Pompe disease is a rare metabolic disease caused by a deficiency of lysosomal enzyme acid-α-glucosidase and characterized by accumulation of glycogen in lysosomes leading to progressive muscle weakening which can result in death due to cardiorespiratory failure (70). Recombinant alglucosidase alfa (GAA) is a life-saving replacement enzyme therapy (71). However, severely deficient patients are prone to develop neutralizing ADAs that comprises activity (72). There is currently no approved rescue therapy for patients that develop ADAs. Kishnani et al. (73) have pioneered the use of methotrexate, rituximab, and IVIG to mitigate the immunogenicity of alglucosidase alfa, which has saved patients' lives.

Joseph et al. (74) showed that transient dosing of methotrexate on days 1, 3, and 5 after each of the first three treatments of GAA also mitigated the ADA response to subsequent challenge injections of GAA alone in a mouse model of Pompe. Recently this finding was translated in a small human clinical trial (75) in Pompe patients that were positive for GAA cross-reactive immunological material (so called CRIM^+^ patients). Because CRIM^+^ patients usually have some level of natural immune tolerance to GAA due to endogenous expression of low levels of GAA or mutant GAA, these patients tend to have less pronounced ADA responses compared to CRIM^−^ patients, and consequently have better clinical responses to GAA therapy (76). Twelve of 14 treatment-naïve CRIM^+^ Pompe patients treated with transient dosing of methotrexate on days 1, 3, and 5 after each of the first three treatments of GAA developed only low titers (<12,800) of anti-GAA antibodies. There was no concurrent control in this pilot clinical study, but the results compare favorably with a retrospective analysis showing the development of high ADA titers in 9 of 23 (39%) CRIM+ patients (77).

The Kishnani group conducted a small pilot study comparing the ability of ImmTOR vs. transient dosing with methotrexate, as described by Joseph et al. (74), for the ability of mitigate the immunogenicity of GAA in a mouse model of Pompe disease (53). ImmTOR treated animals showed more durable inhibition of ADA formation, higher glycogen clearance in skeletal muscles, and improved motor function compared with animals treated with GAA + methotrexate. Moreover, the animals treated with GAA + methotrexate showed a ~5% loss in body weight during the treatment phase, while mice treated with GAA + ImmTOR showed a ~4% gain in body weight over the same period. The body weights of the GAA + methotrexate-treated mice lagged behind those of the GAA + ImmTOR treated mice throughout the duration of the study (10 weeks after the treatment phase). Antibody titers against GAA developed by week 6, after 3 weekly challenges of GAA alone in the GAA + methotrexate-treated animals. In contrast, anti-GAA antibody titers remained low through 10 weeks, after 7 GAA challenge injections, in the GAA + ImmTOR-treated group. However, antibody titers developed by 12 weeks, the last time point measured in the study. These results indicate that tolerance was broken after repeated challenge injections of GAA. It is possible that the durability of tolerance could be extended by additional co-injections of ImmTOR, either at the beginning of therapy or intermittently to reinforce tolerance, analogous to a booster injection used in vaccines (see also section Durability of Tolerance).

### Adeno-Associated Virus

Gene therapy is one of the most promising approaches for the treatment of thousands of rare genetic diseases. The field has experienced a renaissance since the development of AAV as a vector for *in vivo* gene delivery (78). AAV is a non-pathogenic and largely non-integrating virus capable of transducing multiple cell types, including non-dividing cells, but does not induce a strong immune response. However, AAV does elicit the formation of neutralizing antibodies (79, 80). Due to the non-integrating nature of AAV, transgene expression can wane over time due to cell turnover. For many inherited metabolic and degenerative diseases, correction of the defective gene is often needed in infancy or early childhood to limit irreversible progression of disease. However, as the child grows, the target organ, such as the liver, may also increase in mass by several fold. In addition, liver injury, caused by infection or chemicals, may cause further turnover of hepatocytes resulting in further dilution of the transgene. These patients may require retreatment to restore therapeutic benefit. However, currently retreatment is not possible due to the formation of neutralizing antibodies that occur after the initial treatment with AAV vectors. Mitigating the immunogenicity to AAV is particularly challenging because of its size, the repetitive display of antigenic epitopes on the capsid, and the high degree of antibody suppression required to prevent vector neutralization (79, 80).

Mingozzi et al. (48) investigated the ability of ImmTOR to mitigate the formation of anti-AAV antibodies and enable vector re-dosing. In these experiments, animals were transduced with an AAV8 vector expressing an irrelevant transgene on day 0 and then treated with a second AAV8 vector expressing human factor IX on day 21. The rationale behind this design was that expression of the human factor IX transgene should be only be observed if the immune response to the initial dose of AAV was sufficiently inhibited to allow efficient transduction on day 21. These investigators demonstrated that co-administration of ImmTOR with AAV vector prevented the formation of anti-AAV antibodies in both mice and non-human primates and enabled productive expression of the factor IX transgene upon repeat dosing (48). ImmTOR combined with the AAV8 serotype vector did not compromise the immune response to AAV5 serotype, demonstrating antigen selectively to the co-administered capsid. AAV transduction of hepatocytes in the liver appears to be a stochastic process. Using two different transgenes for the first and second administrations, Meliani et al. (48) showed that a second dose of AAV, enabled by the use of ImmTOR, was capable of transducing hepatocytes that were not transduced after the first dose. This may be particularly important for the correction of metabolic diseases of the liver, where the total percentage of transduced cells may be critical for efficacy. Redosing for gene therapy is different from redosing of most biologic therapies, which are typically administered on a regular schedule. In the case of gene therapy, the interval of redosing would likely be a minimum of several months if not years after the initial dose. Meliani et al. reported that optimal mitigation of anti-AAV antibodies required administration of ImmTOR at both the initial and repeat dose of AAV (48). Due to the particulate nature of AAV capsid, which contributes to its immunogenicity, and the fact that even low titers of antibodies can neutralize AAV transduction, the therapeutic dose of SEL-110 for AAV gene therapy applications was typically higher than that required for protein therapies (100–200 μg vs. 50–100 μg). In addition to mitigating the formation of ADAs, ImmTOR treatment inhibited the appearance of CD8 T cells in the liver (48), an event which may be associated with liver inflammation following systemic AAV administration in human patients (81).

### Mitigation of Hypersensitivity Responses

Immune-mediated hypersensitivity reactions are a common cause of adverse events associated with biologic therapies (5). ImmTOR has been shown to inhibit antigen-specific T cell mediated delayed type hypersensitivity reactions, even when administered in the presence of a potent TLR agonist (47). Similarly, ImmTOR was shown to inhibit injection site reactions associated with repeated s.c. injections of adalimumab (47).

Systemic hypersensitivity reactions are more serious and can result in anaphylaxis. Anaphylaxis can be mediated by IgE antibodies that provoke mast cell activation or by IgG immune complexes that can result in complement activation and myeloid cell activation. ImmTOR co-administered with ovalbumin has been shown to inhibit the formation of antigen-specific IgE antibodies and IgE-mediated allergic reactions (34). Repeated high doses of KLH administered i.v. induced the formation of high titer IgG antibodies that led to anaphylaxis in animals. Co-administration of ImmTOR with KLH inhibited both antibody formation and the anaphylactic response (47).

### Pre-existing Immunity

Mitigating or reversing pre-existing immunity is challenging, particularly for pre-existing antibodies. One of the salient features of the adaptive immune system is the formation of memory T and B cells that enable rapid and robust anamnestic responses and the formation of long-lived plasma cells (LLPCs) that continue to produce antibodies even in the absence of further antigen stimulation (52).

For emerging antibody responses against coagulation FVIII, low titer antibodies induced by 3–6 injections of FVIII alone could be reduced in the majority of animals by repeated therapeutic treatment with FVIII + ImmTOR (45). Mazor et al. (35) induced low levels of ADAs against recombinant immunotoxin and rested animals for 6 weeks to allow memory cells to form. Challenging the mice with immunotoxin alone induced a massive anamnestic response resulting in titers that were approximately ten times higher. However, therapeutically treating the animals with ImmTOR + immunotoxin not only prevented the boost in antibody titer, but actually further reduced titers close to baseline levels. In the presence of high titer antibodies (>10,000), induced with 12 injections of recombinant immunotoxin, ImmTOR + immunotoxin could reduce titers 5–10-fold (35). However, even a 5–10-fold reduction in high antibody titers may still affect the activity or pharmacokinetics of a biologic therapy. Thus, the ability of ImmTOR to mitigate pre-existing antibody titers may vary with the antigen and the level of pre-existing antibodies. For T cell-mediated disease, such as EAE, a single dose of ImmTOR containing both rapamycin and PLP antigen administered at the peak of disease was sufficient to resolve disease symptoms and prevent disease relapse (34, 48).

### Durability of Tolerance

There are two types of Foxp3+ Tregs (82). Natural Tregs (nTregs) are selected in the thymus based on their reactivity to self-antigens and are critical to maintain tolerance to self. However, naïve T cells that are weakly reactive to self-antigen can escape the thymus and have the potential to become self-reactive. Adaptive Tregs (aTreg) can be induced in the periphery to limit autoimmune responses. The aTreg are also critical for the induction of tolerance to beneficial commensal bacteria, food antigens and harmless environmental antigens. Immune tolerance to biologic agents can leverage nTreg in the case of replacement enzyme or protein therapies, such as FVIII or aglucosidase alpha. However, patients that are completely deficient in the expression of the endogenous protein may lack nTreg specific to the protein and thus are more likely form ADAs (72). Immune tolerance induction in such patients may require induction of aTregs. Similarly, induction of aTreg are critical for biologics that are foreign to the human immune system, like uricase or AAV. The aTreg are more plastic than nTreg and may become unstable in certain inflammatory conditions (82). This plasticity is important in the event that a “harmless” microbe becomes pathogenic. Thus, induction of immune tolerance is not an irreversible on-off switch. Rather maintenance of tolerance is a dynamic process between pro-tolerogenic and pro-stimulatory signals. The ratio of Treg to effector T cells can determine the outcome of immune tolerance vs. immune stimulation (29). One of the key outstanding questions in the translation of immune tolerance technologies is the durability of aTreg-mediated tolerance.

The durability of tolerance to a biologic therapy may be impacted by a number of factors, including drug-related properties and patient or disease-related factors (83–85). The inherent immunogenicity of the biologic drug can impact the durability of tolerance, as repeated challenge with highly immunogenic antigens could provide an overwhelming immunostimulatory bias (5, 6). Key factors that promote immunogenicity are repetitive display of antigenic-epitopes (e.g., multimeric proteins), the propensity to form micro-aggregates, dose route and regimen, antigens that cause tissue damage or inflammation, and the absence of natural tolerance (e.g., proteins that are foreign to the immune system). Patient and disease-specific factors may include an inflammatory milieu, pre-existing immunity, immune status, co-medications, and genetics. The age of the patient may also be a factor, as the production of naïve T cells wanes with the involution of the thymus, and the T cell repertoire becomes comprised primarily of antigen-experienced memory T cells (86, 87).

Preclinical studies have shown the ability of ImmTOR to induce durable tolerance to a variety of highly immunogenic proteins that withstands multiple challenges with antigen alone. For KLH, five s.c. co-administrations of KLH + ImmTOR maintained tolerance for at least 5 months during which animals were challenged 11 times with KLH alone (47). Similarly, for adalimumab, 7 co-treatments enabled tolerance that was maintained after 9 challenge injections (47). For coagulation FVIII, 5 combination treatment provided sustained mitigation of ADAs for at least 5 months after treatment (45), and for recombinant immunotoxin, two cycles of treatment induced tolerance that allowed for three additional cycles (9 injections) of immunotoxin alone (35). Finally, for recombinant alglucosidase alpha, 3 weekly combination treatments mitigated the formation of ADAs for 7 challenge injections; however, ADA developed by the time of the 10th challenge injection (53). Immune tolerance is a dynamic process balancing pro-stimulatory and pro-tolerogenic signals, and can be broken by repeated injections of a highly immunogenic antigen. It is possible that additional co-treatments with ImmTOR may be required for more durable tolerance or that periodic retreatment with ImmTOR might be needed to reinforce immune tolerance. However, the number and timing of such additional treatments may need to be determined empirically for each biologic and disease setting. This may be a key challenge for successful clinical translation of applications in which patients require life-long therapy, such as the case for GAA in Pompe disease or FVIII in hemophilia A.

### Clinical Translation

Most of the preclinical studies with ImmTOR have been performed in inbred strains of laboratory mice, which have their obvious limitations with respect to their anatomy, immune system, genetic diversity, lifespan, microbiome, and environmental factors for translating findings to humans. Oral tolerance therapies for autoimmune disease that looked promising in animal studies have not translated to humans (88). While there are many promising immune tolerance technologies and strategies on the horizon (15–21), translation to humans remains a formidable challenge. Applying immune tolerance strategies to autoimmune diseases adds a layer of risk due to heterogenous disease presentation and progression, antigen uncertainty, generally poor animal models, and the requirement to reverse a well-established immune response. We have tried to mitigate some of this risk by focusing initially on mitigation of ADAs to biologic therapies, which has the advantage of a well-defined antigen, a robust biomarker readout (ADA levels), and the ability to treat prophylactically.

The ability of ImmTOR to mitigate the formation of ADAs in human has been evaluated in combination with pegadricase, a highly immunogenic, pegylated uricase enzyme of fungal origin, in patients with hyperuricemia. A Phase 1b single ascending dose, open-label, multi-center clinical trial (NCT02648269) conducted in the United States showed a dose-dependent inhibition of uricase-specific ADAs (89). The activity of pegadricase was monitored through the measurement of serum uric acid (SUA). In gout, the therapeutic goal is to lower SUA levels below 6 mg/dL, as higher levels can result in the deposition of urate crystals in joints and soft tissues. Patients were selected for baseline SUA >6 mg/dL. All patients treated with enzyme alone showed an initial drop in serum uric acid (SUA) levels that was maintained for the first week after treatment. However, by day 14, SUA levels started to rebound and by day 30, 4 of 5 patients were back to baseline levels of SUA. All patients treated with a single dose of pegadricase alone developed high titers of ADAs by day 14, which correlated with rapid clearance of serum uricase activity. The addition of ImmTOR showed a dose-dependent inhibition of ADA formation. Mitigation of ADAs correlated with prolonged pharmacodynamic activity of pegadricase and sustained reduction in sUA levels for at least 30 days after a single dose (89). These results suggest that combination of ImmTOR + pegadricase would support monthly dosing. SEL-110 was generally well-tolerated at doses up to 0.3 mg/kg. No deaths or life-threatening treatment emergent adverse events (TEAEs) were reported during the study, and overall, there were no notable trends in the nature or frequency of TEAEs. There were no clinically significant changes in clinical laboratory values, vital signs or ECGs during the course of the study. Interim data from a Phase 2 multidose, open-label, multi-center clinical trial (NCT02959918) indicate that multiple monthly doses of SEL-212, the combination of ImmTOR + pegadricase, is able to maintain SUA <6 mg/dL in the majority of patients (61).

## Conclusion

The full impact of ADAs on healthcare is largely unknown, as ADAs are not routinely measured after drug approval due to the lack of effective ADA mitigation strategies. However, patients that develop ADAs may experience disease progression due to ADAs that compromise efficacy and may be exposed to an increased risk of adverse events (5, 6). ADAs also place a burden on healthcare costs (5). In addition, there are opportunity costs related to the late-stage abandonment of promising but immunogenic biologic drugs in the pipeline (10, 11). While companies strive to minimize immunogenicity during development on a product-specific basis, there is a need for an approach to ADA mitigation that can be applied broadly across many types of biologic therapies. The use of ImmTOR nanoparticles is a promising approach to mitigate the immunogenicity of a diverse array of biologics without the need to reformulate or alter the biologic therapy. Treatment with ImmTOR induces dendritic cells with a tolerogenic phenotype and regulatory T cells specific to the co-administered biologic therapy resulting in inhibition of T and B cell activation and ADA formation. Early clinical studies of SEL-212, a combination product of ImmTOR + a pegylated uricase enzyme, provide proof-of-concept for ADA mitigation against a highly immunogenic enzyme in humans. ImmTOR has the potential to improve the efficacy and safety of biologic therapies for patients and warrants further study.

## Author Contributions

TK wrote the manuscript.

## Conflict of Interest

TK is an employee and shareholder of Selecta Biosciences, and ImmTOR is being developed by Selecta Biosciences.
